# Inter-row information recognition of maize in the middle and late stages *via* LiDAR supplementary vision

**DOI:** 10.3389/fpls.2022.1024360

**Published:** 2022-12-01

**Authors:** Zhiqiang Li, Dongbo Xie, Lichao Liu, Hai Wang, Liqing Chen

**Affiliations:** ^1^ College of Engineering, Anhui Agricultural University, Hefei, China; ^2^ Anhui Intelligent Agricultural Machinery Equipment Engineering Laboratory, Hefei, China; ^3^ Discipline of Engineering and Energy, Murdoch University, Perth, WA, Australia

**Keywords:** inter-row information recognition, point cloud, maize plant protection, lidar, machine vision

## Abstract

In the middle and late stages of maize, light is limited and non-maize obstacles exist. When a plant protection robot uses the traditional visual navigation method to obtain navigation information, some information will be missing. Therefore, this paper proposed a method using LiDAR (laser imaging, detection and ranging) point cloud data to supplement machine vision data for recognizing inter-row information in the middle and late stages of maize. Firstly, we improved the YOLOv5 (You Only Look Once, version 5) algorithm based on the characteristics of the actual maize inter-row environment in the middle and late stages by introducing MobileNetv2 and ECANet. Compared with that of YOLOv5, the frame rate of the improved YOLOv5 (Im-YOLOv5) increased by 17.91% and the weight size decreased by 55.56% when the average accuracy was reduced by only 0.35%, improving the detection performance and shortening the time of model reasoning. Secondly, we identified obstacles (such as stones and clods) between the rows using the LiDAR point cloud data to obtain auxiliary navigation information. Thirdly, the auxiliary navigation information was used to supplement the visual information, so that not only the recognition accuracy of the inter-row navigation information in the middle and late stages of maize was improved but also the basis of the stable and efficient operation of the inter-row plant protection robot was provided for these stages. The experimental results from a data acquisition robot equipped with a camera and a LiDAR sensor are presented to show the efficacy and remarkable performance of the proposed method.

## 1 Introduction

Maize is one of the five most productive cereals in the world (the other four being rice, wheat, soybean, and barley) (Patricio and Rieder, 2018) that is an important source of food crops and feed. In recent years, with the rapid increase in maize consumption, an efficient and intelligent maize production process has been required to increase productivity (Tang et al., 2018; Yang et al., 2022a). Inter-row navigation is a key to realizing the intelligence of maize planting. Pest control in the middle and late stages of maize determines the crop yield and quality. A small autonomous navigation plant protection robot is a good solution for plant protection in the middle and late stages of maize development (Li et al., 2019). However, in these stages, the high plant height (Chen et al., 2018), insufficient light, and several non-maize obstacles lead to a typical high-occlusion environment (Hiremath et al., 2014; Yang et al., 2022b). Commonly used navigation systems such as GPS (Global Positioning System) and BDS (BeiDou Navigation Satellite System) have shown poor signal quality in a high-occlusion environment (Gai et al., 2021); therefore, accurately obtaining navigation information between rows in the middle and late stages of maize has become the key issue to realizing the autonomous navigation of plant protection robots. At present, machine vision is the mainstream navigation method used to obtain inter-row navigation information in a high-occlusion environment (Radcliffe et al., 2018); that is, the RGB (red, green, and blue) camera acquires images of the maize stems, identifies maize stems through a trained model, and obtains position information so as to plan the navigation path. The convolutional neural network was used to train the robot to recognize the characteristics of maize stalks at the early growth stage, which was implemented on an inter-row information collection robot based on machine vision (Gu et al., 2020). Tang et al. reported the application and research progress of harvesting robots and vision technology in fruit picking (Tang et al., 2020). The authorsMachine vision technology was applied for the multi-target recognition of bananas and automatic positioning for the inflorescence axis cutting point (Wu et al., 2021); in addition, the improved YOLOv4 (You Only Look Once, version 4) micromodel and binocular stereo vision technology were applied for fruit detection and location (Wang et al., 2022; Tang et al., 2023). Zhang et al. proposed an inter-row information recognition algorithm for an intelligent agricultural robot based on binocular vision, where the effective inter-row navigation information was extracted by fusing the edge contour and height information of crop rows in the image (Zhang et al., 2020). By setting the region of interest, Yang et al. used machine vision to accurately identify the crop lines between rows in the early growth stage of maize and extracted the navigation path of the plant protection robot in real time (Yang et al., 2022a). However, the inter-row environment in the middle and late stages of maize is a typical high-occlusion environment, with higher plant height and dense branches and leaves, seriously blocking light (Liu et al., 2016; Xie et al., 2019). When the ambient light intensity is weak, information loss will occur when using machine vision to obtain inter-row navigation information (Chen et al., 2011). However, considering the fact that machine vision usually takes a certain feature of maize as the basis for the acquisition of information, recognizing multiple features at the same time will greatly reduce the recognition speed and also reduce the real-time performance of agricultural robots, taking non-maize obstacles into consideration (such as soil, bricks, and branches) in the middle and late stages of maize; it is, therefore, quite difficult to obtain all the inter-row information by using only a single feature.

Since LiDAR (laser imaging, detection and ranging) can obtain accurate point cloud data of objects according to the echo detection principle (Reiser et al., 2018; Wang et al., 2018; Jafari Malekabadi et al., 2019) and is less affected by light (Wang et al., 2022a; Wang et al., 2022b), it can supplement the missing information caused by the use of machine vision (Jeong et al., 2018; Aguiar et al., 2021). In order to solve the issue of information loss when a vision sensor was used to obtain information, a method using LiDAR supplement vision was proposed (Bae et al., 2021), which pooled the strength of each sensor and made up for the shortcomings of using a single sensor. Through the complementary process between vision and LiDAR (Morales et al., 2021; Mutz et al., 2021), the performance of adaptive cruise control was significantly improved; thus, a complementary method combining vision and LiDAR was developed in order to further improve the accuracy of unmanned aerial vehicle (UAV) navigation (Yu et al., 2021). Liu et al. proposed a new structure of LiDAR supplement vision in an end-to-end semantic segmentation network, which can effectively improve the performance of automatic driving (Liu et al., 2020). The above methods had good application effects in the field of autonomous driving (Chen et al., 2021; Yang et al., 2021; Zhang et al., 2021). Based on the above research, we believe that LiDAR supplement vision is an interesting and effective method to obtaining inter-row information in the middle and late stages of maize development.

Therefore, this paper proposed a method of using LiDAR point cloud data to supplement machine vision data for obtaining inter-row information in the middle and late stages of maize. We took the location of maize plants as the main navigation information and proposed an improved YOLOv5 (Im-YOLOv5) algorithm (Jubayer et al., 2021, p. 5) to identify maize plants and obtain the main navigation information. At the same time, we took the locations of stones, clods, and other obstacles as auxiliary navigation information, which were obtained through LiDAR. By the supplementary function of vision and LiDAR, the accuracy of the inter-row navigation information acquisition in the middle and late stages of maize can be improved. The proposed method provides a new and effective way to obtaining navigation information between rows in the middle and late stages of maize under the condition of equal height occlusion.

The contributions of this article are summarized as follows:

1. A method of inter-row information recognition with a LiDAR supplement camera is proposed.
2. An Im-YOLOv5 model with efficient channel attention (ECA) and lightweight backbone network is established.
3. Auxiliary navigation information acquisition using LiDAR can reduce the loss of information.
4. The proposed method was tested and analyzed using a data acquisition robot.

## 2 Methods and materials

### 2.1 Composition of the test platform

**Figure 1:**
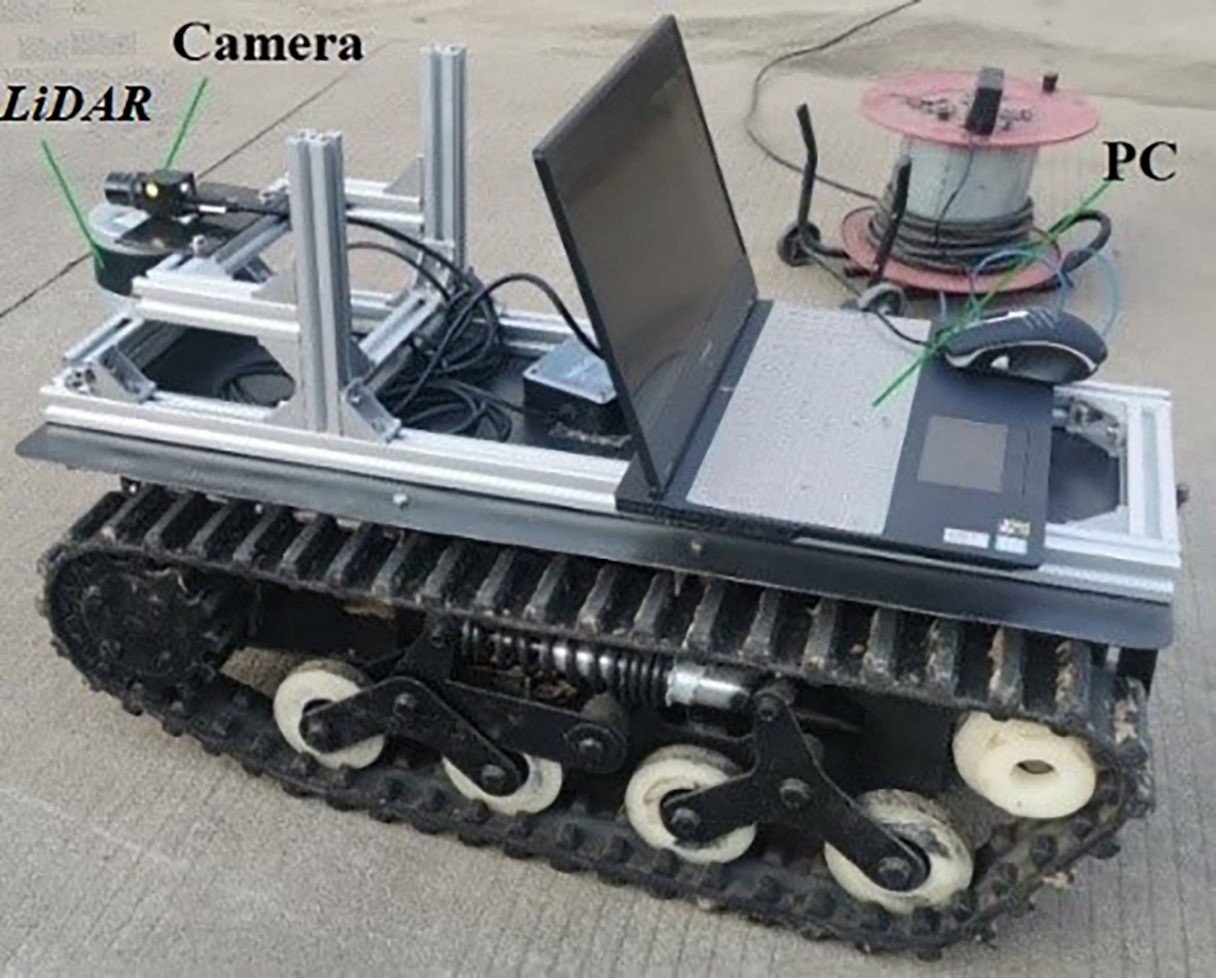
Data acquisition robot. *PC*, personal computer.

The experimental platform and data acquisition system are shown in **Figure 1**. A personal computer (PC) was used as the upper computer to collect LiDAR and camera signals. The LiDAR model is VLP-16, the scanning distance was 100 m, the horizontal scanning angle was 270°, and the vertical scanning angle was ±15°. The camera model is NPX-GS650, the resolving power was 640*480, and the frame rate was 790.

### 2.2 Commercialization feasibility analysis

The data acquisition platform used in the test costs 490 RMB. The plant protection operation can be carried out by installing a pesticide applicator in the later stage, with the cost of the pesticide applicator about 100 RMB. The cost of the camera sensor was about 100 RMB, and that of the LiDAR sensor was about 5,000 RMB. Consequently, the cost of VLP-16 LiDAR represented a key issue affecting the commercialization of this recognition system. Therefore, our recognition system was applied to small autonomous navigation plant protection robots. The relatively low-cost of small plant protection robots, even with the application of this relatively high-precision recognition system, had a price advantage over UAVs.

## 3 Joint calibration of camera and LiDAR

In this paper, a monocular camera and VLP-16 LiDAR were used as the information fusion sensors. When the monocular camera and the LiDAR detect the same target, despite the range and angle information being the same, the detection results of the two sensors belong to different coordinate systems (Chen et al., 2021a). Therefore, in order to effectively realize the information supplementation of LiDAR to the camera, the coordinate system must be unified; that is, the detection results of the two sensors should be input into the same coordinate system and the relative pose between them should be calibrated at the same time so as to realize the data matching and correspondence between these two sensors.

**Figure 2:**
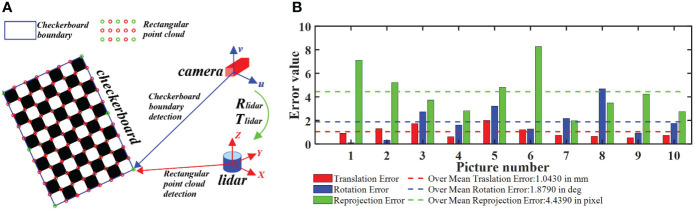
Camera–LiDAR (laser imaging, detection and ranging) joint calibration process. **(A)** Principle of joint calibration. **(B)** Joint calibration error. By matching the corner information of the chessboard picture taken by the camera to the corner information of the chessboard point cloud data obtained by LiDAR, the rigid transformation matrix from the point cloud data to the image can be obtained.

It should be noted that the main task of the monocular camera calibration was to solve its extrinsic parameter matrix and intrinsic parameters. In this paper, the chessboard calibration method was used (Xu et al., 2022), with the chessboard size being 400 mm × 550 mm and the grid size being 50 mm ×  50; mm. We randomly took 21 chessboard pictures of different positions. The camera calibration error was less than 0.35 pixels and the overall mean error was 0.19 pixels, which means, according to reference, that the error met the calibration accuracy and that the calibration result has practical value (Xu et al., 2021). The internal parameters of the camera were as follows: focal length (*f*) = 25 mm, radial distortion parameter (*k*_1_) = 0.012 mm, radial distortion parameter (*k*_2_) = 0.009 mm, tangential distortion parameter (*p*_1_) = −0.0838 mm, tangential distortion parameter (*p*_2_) = 0.1514 mm, image center (*u*_0_) = 972 mm, image center (*v*_0_) = 1,296 mm, normalized focal length (*f_x_* = *f/dx*) = 1,350.3 mm, and normalized focal length (*f_y_* = *f/dy*) = 2,700.8 mm. On the basis of camera calibration, we carried out the joint calibration of the camera and LiDAR. The calibration principle is shown in **Figure 2A**. By matching the corner information of the chessboard picture taken by the camera to the corner information of the chessboard point cloud data obtained by LiDAR, a rigid transformation matrix from the point cloud data to the image can be obtained. During calibration, the camera and LiDAR were fixed on the data acquisition robot platform developed by the research group. After the joint calibration, the relative positions of the camera and LiDAR were saved and fixed. The calibration error is shown in **Figure 2B**. As indicated in Aguiar et al. (2021), the calibration error met the calibration accuracy, and the calibration result showed practical value. Through joint calibration, the rigid transformation matrix of the point cloud projection to the image is obtained from Equations (1) and (2).


(1) $$R_{\mathrm{lidar}}=\left[\begin{array}{c} 0.9998\\ -0.0032\\ 0.0179 \end{array}\begin{array}{c} 0.0176\\ -0.0807\\ -0.9966 \end{array}\begin{array}{c} 0.0047\\ -0.9967\\ 0.0806 \end{array}\right]$$

(2) $$T_{\mathrm{lidar}}=\left[\begin{array}{c} 0.0468\\ 0.1139\\ -0.2667 \end{array}\right]$$

## 4 Navigation information acquisition based on LiDAR supplement vision

**Figure 3:**
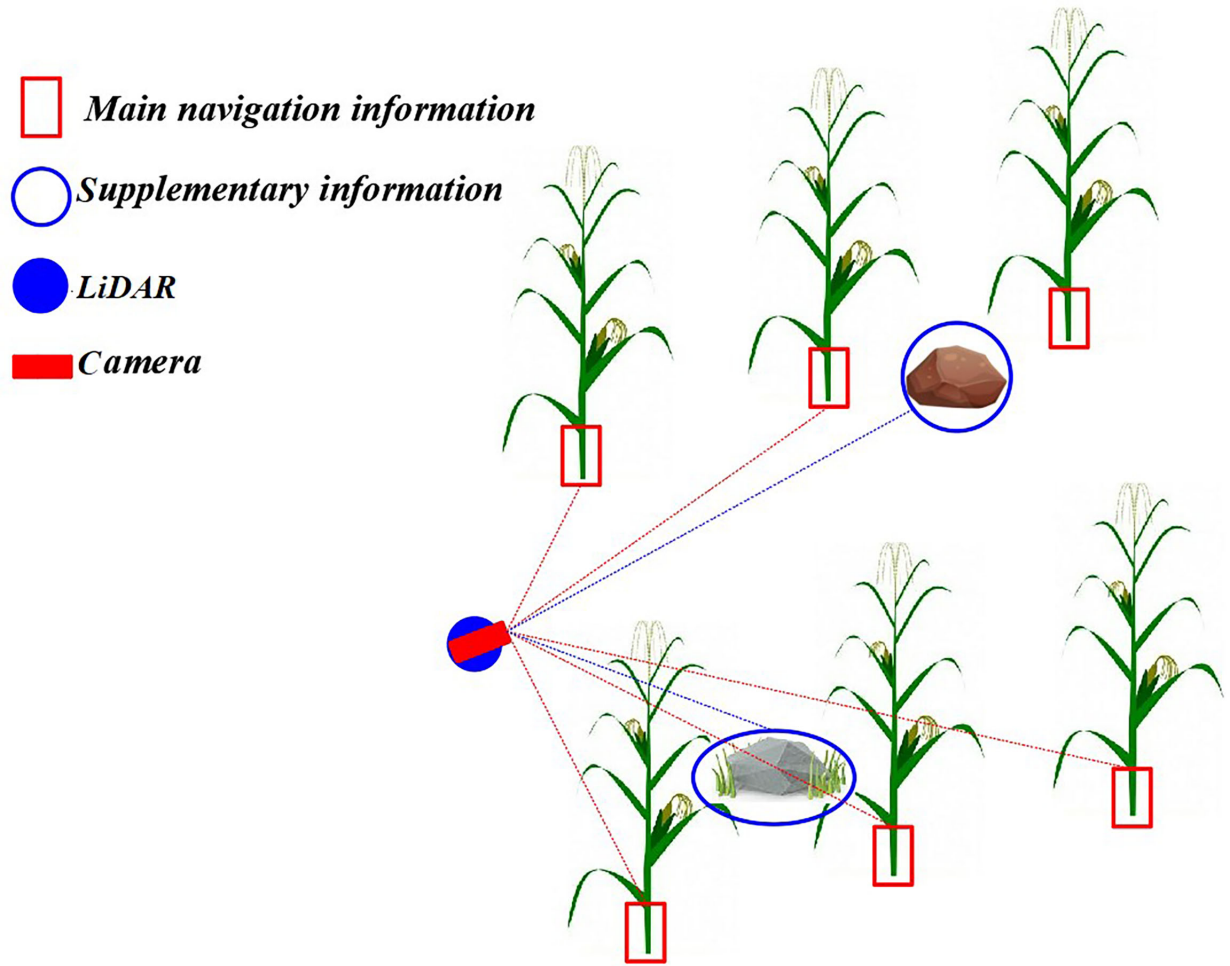
Principle of navigation information acquisition based on LiDAR (laser imaging, detection, and ranging) supplement camera. The machine vision datasets were trained using the improved YOLOv5 (Im-YOLOv5) algorithm to identify the stem of the maize and then obtain the main navigation information, while LiDAR was used to obtain auxiliary navigation information.

As mentioned in Section 1, machine vision usually takes a single feature of the plant as the basis of recognition. In this paper, the maize stem about 10 cm above the ground surface was taken as the machine vision recognition feature. It should be noted that taking the maize stem as the identification feature will cause lack of information on the other non-maize obstacles (such as stones and clods). In order to solve the issue of missing information when using machine vision to acquire navigation information, this paper proposed a method of inter-row navigation information acquisition in the middle and late stages of maize based on LiDAR supplement vision. The detailed principle is shown in **Figure 3**. The machine vision datasets were trained using the Im-YOLOv5 algorithm to identify the stem of the maize and, subsequently, to obtain the main navigation information. The point cloud data of the inter-row environment in the middle and late stages of maize were obtained using LiDAR to gather auxiliary navigation information. It should be noted that the method proposed in this paper obtained inter-line information through LiDAR-assisted cameras; therefore, spatial data fusion was used. After establishing the precise coordinate conversion relationship among the radar coordinate systems—a three-dimensional world coordinate system—a camera coordinate system, an image coordinate system, and a pixel coordinate system—the spatial position information of the obstacles in the point cloud data can be matched to the visual image.

### 4.1 Main navigation information acquisition with the improved YOLOv5

YOLO models have a real-time detection speed, but require a powerful GPU (graphic processing unit) and a large amount of memory when training, limiting their use on most computers. The large size of the model after training can also increase the hardware requirements on mobile devices. Ideally, a detection model would meet the requirements of detection accuracy and real-time detection speed of maize stems, without high hardware requirements. The YOLOv5 model is a lightweight version of YOLO, has fewer layers and faster detection speed, can be used on portable devices, and requires fewer GPU resources for training (Tang et al., 2023). Therefore, the goal of this work was to build on the YOLOv5 model and apply the improved model for the detection of maize stems. The main idea for improving YOLOv5 was to lighten its backbone network through MobileNetv2 and introduce the ECANet attention mechanism to improve the recognition accuracy and robustness of the model.

#### 4.1.1 Lightweight Backbone network

This paper used MobileNetv2 (Zhou et al., 2020) to replace the backbone network of YOLOv5 for the extraction of maize stem images with effective characteristics. In order to enhance the adaptability of the network to the task of recognizing maize stem features and fully extract features, a progressive classifier was designed in this paper to enhance the network’s recognition ability of the corn rhizome. The original MobileNetV2 network was primarily used to deal with more than 1,000 types of targets on the ImageNet dataset, while this paper only targeted maize stems. Therefore, in order to better extract the characteristics of maize stems and improve the recognition ability of the network on maize stems, we the classifier of the network was redesigned, which included two convolution layers, one global pooling layer, and one output layer (convolution layer).

**Figure 4:**
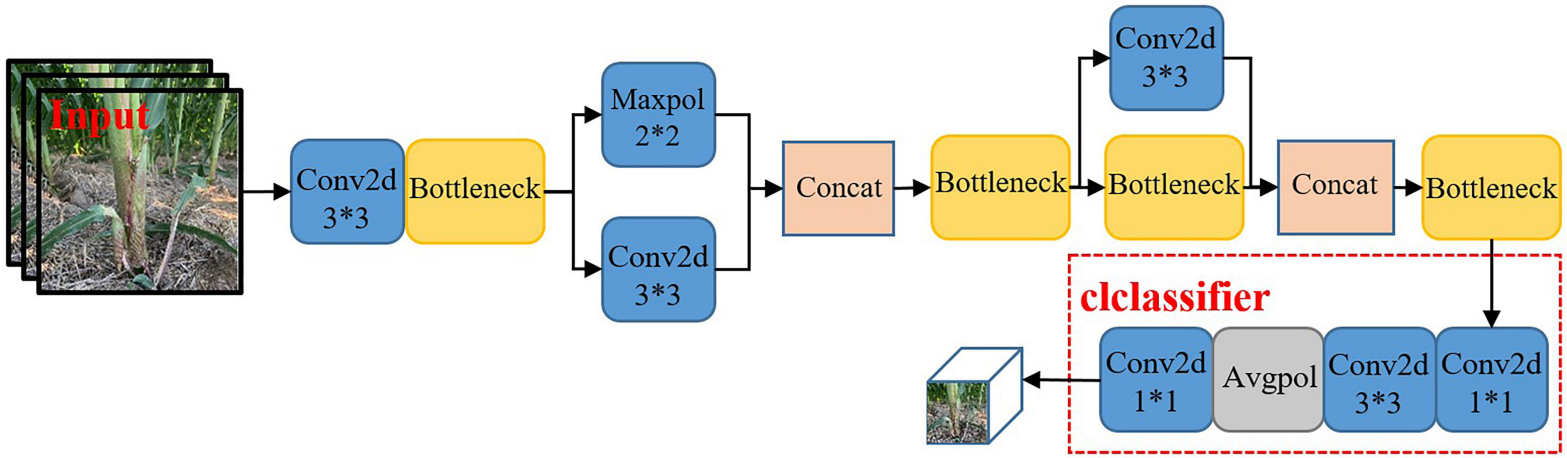
MobileNetv2 network structure.

The main task of the classifier was to efficiently convert the extracted maize stem features into specific classification results. As shown in **Figure 4**, two convolution kernels with different scales were selected to replace a single convolution kernel in the original classifier in order to perform the compression and conversion operations of the feature map. The size of the first convolution kernel was 1 × 1. It was mainly responsible for the channel number compression of the feature map. In order to avoid the loss of a large number of useful features caused by a large compression ratio, the second convolution was used mainly for the size compression of the feature map to avoid fluctuations in the subsequent global pooling on a large feature map. Comparison of the Im-YOLOv5 network based on MobileNetv2 with the original YOLOv5 network showed that the model parameters decreased from 64,040,001 to 39,062,013 and the parameters decreased by 39%.

At the same time, Im-YOLOv5 used CIOU_Loss [complete intersection over union (IOU) loss] to replace GIOU_Loss (generalized IOU loss) as the loss function of the bounding box and used binary cross-entropy and logits loss function to calculate the loss of class probability and target score, defined as follows.


(3) $$GIOU=IOU-\left|\frac{C-(A\cup^{}B)}{\left|C\right|}\right|$$

(4) $$IOU=\frac{\left|A\cap^{}B\right|}{\left|A\cup^{}B\right|}$$

(5) $$CIOU=IOU-\frac{\rho^{2}(b,b^{gt})}{c^{2}}-a\nu$$

In Equations (3) and (4), *A* and *B* are the prediction box and the real box, respectively; IOU is the intersection ratio of the prediction box and the real box; and *C* is the minimum circumscribed rectangle of the prediction box and the target box. However, Equations (3) and (4), considering only the overlap rate between the prediction box and the target box, cannot describe well the regression problem of the target box. When the prediction box is inside the target box and the size of the prediction box is the same, GIOU will degenerate into IOU, which cannot distinguish the corresponding positions of the prediction box in each target box, resulting in error detection and leak detection. Equation (5) is the calculation formula of CIOU, where *a* = *v*/(1-IOU)*v* is an equilibrium parameter that does not participate in gradient calculation; v = 4/π^2(arctan (*W^gt^/H^gt^*) – arctan (*W/H*))^2^ is a parameter used to measure the consistency of the length-width ratio; *b* is the forecast box; *b^gt^* is the realistic box; *ρ* is the Euclidean distance; and *c* is the diagonal length of the minimum bounding box. It can be seen from Equation (5) that the CIOU comprehensively considers the overlapping area, center point distance, aspect ratio, and other factors of the target and prediction boxes and solves the shortcoming of the GIOU loss function, making the regression process of the target box more stable, with faster convergence speed and higher convergence accuracy.

#### 4.1.2 Introducing the attention mechanism

**Figure 5:**
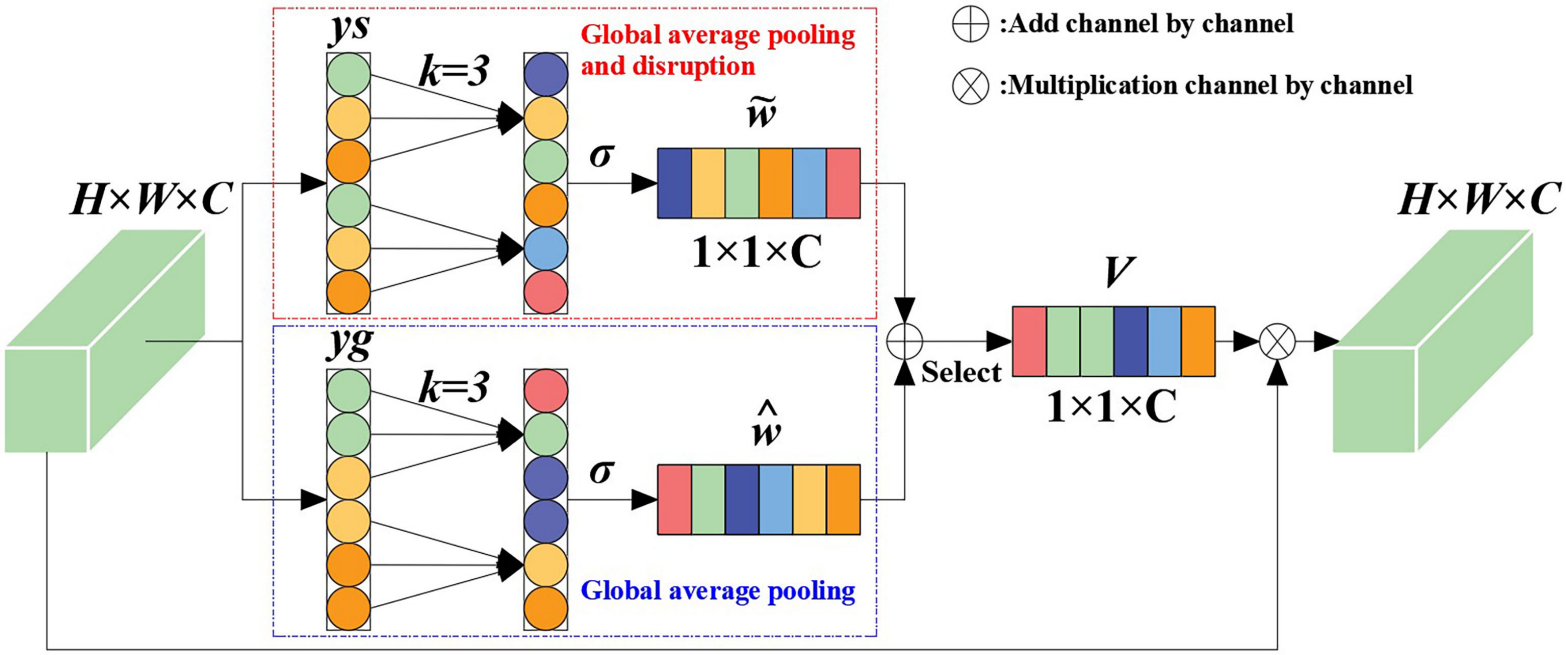
ECANet channel attention.

In order to improve the recognition accuracy and robustness of the algorithm in the case of a large number of maize stems and mutual occlusion between stems, efficient channel attention (ECA) was introduced (Xue et al., 2022). It should be noted that, although the introduction of ECANet into convolutional neural networks has shown better performance improvements, ECANet only considers the local dependence between the current channel of the feature map and several adjacent channels, which inevitably loses the global dependence between the current channel and other long-distance channels. On the basis of ECANet, we added a new branch (shown in the dashed box in **Figure 5**) that has undergone channel-level global average pooling and is disrupted. This branch randomly rearranges the channel order of the feature map after undergoing channel-level global average pooling, so the long-distance channel before disruption may become its adjacent channel. After obtaining the local dependencies between the current channel of the new feature map and its new *k* adjacent channels, weighting the two branches can obtain more interaction information between channels.

In this paper, suppose that the feature vector of the input feature after convolution is *x ϵ R^W×H×C^*, where *W*, *H*, and *C* respectively represent the width, height, and channel size of the feature vector. The global average pooling of the channel dimension can be expressed as:


(6) $$y=g(x)=\frac{1}{WH}\sum_{i=1,j=1}^{W,H}x_{ij}$$

Then, in ECANet, the feature vector inputs by the two branches can be expressed as:


(7) $$ys=S(g(x))=S\left(\frac{1}{WH}\sum_{i=1,j=1}^{W,H}x_{ij}\right)$$

(8) $$yg=g(x)=\frac{1}{WH}\sum_{i=1,j=1}^{W,H}x_{ij}$$

where *ys* represents the vector obtained after global average pooling and disrupting the sequential branching of channels; *yg* represents the vector obtained after global average pooling and branching; and *S* is a channel-disrupting operation. Given that the feature vector without dimension reduction is *y ϵ R^C^*, the inter-channel weight calculation using the channel attention module can be expressed as:


(9) $$\omega =\sigma (W_{k}y)$$

where *σ*(*x*) = 1/(1+*e^-x^*) is the sigmoid activation function and *W_k_* is the parameter matrix for calculating channel attention using ECANet.

We took MobileNetv2 (Zhou et al., 2020) as the backbone model, combined YOLOv5 with the SeNet and ECANet modules (Hassanin et al., 2022), and carried out maize stem recognition experiments. The test results are shown in **Table 1**. ECANet showed better performance compared toSeNet, indicating that ECANet can improve the performance of YOLOv5 with less computational costs. At the same time, ECANet was more competitive than SeNet, and the model complexity was also lower.

**Table 1:** Comparison of the recognition performance (in percent) of the YOLOv5 model integrating different attention modules.

Method	*P*	*R* (%)	FPS (%)	F_1_ (%)	mAP (%)
MobileNetv2	89.5	94.1	34.2	93.7	91.37
MobileNetv2+SeNet	93.2	91.4	63.7	91.2	97.25
MobileNetv2+ECANet	96.7	82.3	79.6	86.3	96.98

P, comparison of accuracy; R, recall; F_1_, harmonic average; FPS, frame rate; mAP, mean average precision.

**Figure 6:**
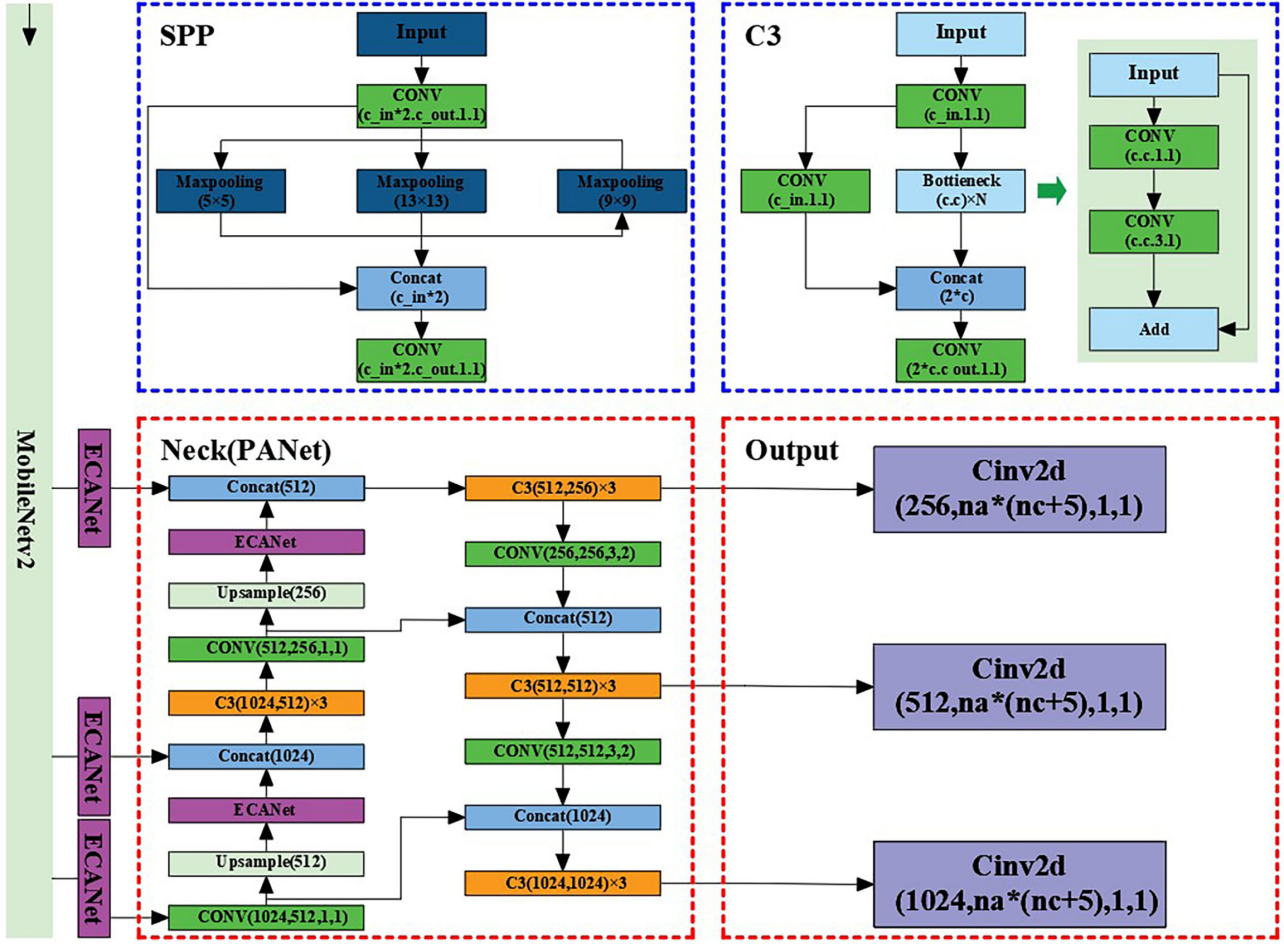
Improved YOLOv5 (Im-YOLOv5) architecture.

In this work, the ECANet attention mechanism was first placed on the enhanced feature extraction network and the attention mechanism added on the three effective feature layers extracted from the backbone network. Regarding the problems of information attenuation, the aliasing effect of cross-scale fusion and the inherent defects of channel reduction in the feature pyramid network (FPN) in YOLOv5, in this paper, we added the ECANet attention mechanism to the sampling results on FPN in order to reduce information loss and optimize the integration characteristics on each layer. By introducing the ECANet attention mechanism, Im-YOLOv5 can better fit the relevant feature information between the target channels, ignore and suppress useless information, and make the model focus more on training the specific category of maize stems, strengthening it and improving its detection performance. The specific structure of the Im-YOLOv5 algorithm is shown in **Figure 6**.

### 4.2 Auxiliary navigation information acquisition by LiDAR

Because of the obvious color and structural characteristics of maize stems, we trained the Im-YOLOv5 model to only detect maize stems when the main navigation information was obtained through machine vision. However, the actual non-maize obstacles were mainly soil blocks and stones, and the color and shape characteristics of such obstacles are relatively close to the ground color, which greatly increased the difficulty of Im-YOLOv5 model training. At the same time, recognizing multiple features simultaneously by machine vision will also reduce the recognition speed to a certain extent. Under this condition, it is necessary to obtain point cloud information using LiDAR to supplement machine vision.

#### 4.2.1 Determination of the effective point cloud range

**Figure 7:**
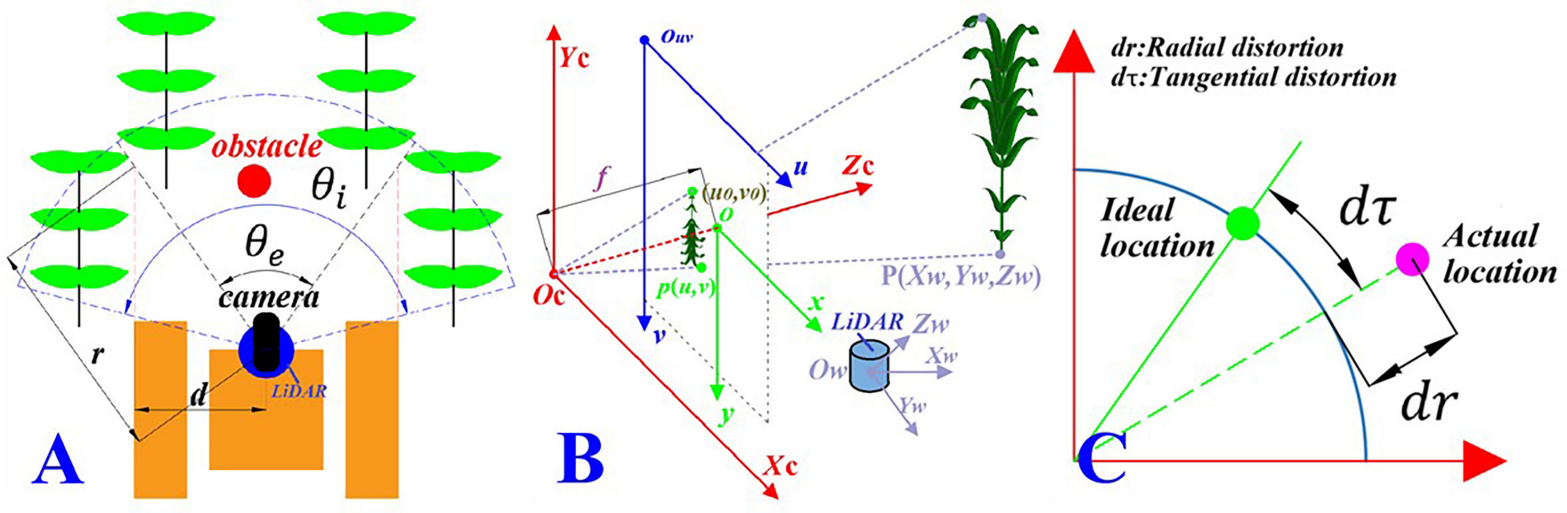
Camera–LiDAR (laser imaging, detection, and ranging) joint calibration process. **(A)** Effective data range. *θ_e_* is the camera shooting angle range, *θ_i_* is the scanning angle of LiDAR, and the overlapping area is the effective point cloud range. **(B)** Coordinate transformation. *O_w_ - X_w_Y_w_Z_w_* is the LiDAR coordinate system, *O_c_ – X_c_Y_c_Z_c_* is the camera coordinate system, o - *xy*is the image coordinate system, and *O_uv_ – uv* is the pixel coordinate system. **(C)** Distortion error. *dr* and dτ are the radial distortion and the tangential distortion of the camera, respectively.

Since the camera and LiDAR were fixed on the data acquisition robot platform, when the robot is walking between lines during data acquisition, it is necessary to determine the effective data range of the LiDAR point cloud according to the shooting angle range of the camera, as shown in **Figure 7A**.

Note that, in **Figure 7A**, *θ_e_* is the camera shooting angle range, *θ_e_* is the scanning angle of LiDAR, and *d* is the width of the robot. Therefore, the range of the effective point cloud data collected by LiDAR is the sector area, where *r* is the radius of the sector with the angle of *θ_e_* and is defined as:


(10) $$r=\frac{d}{\cos\left(\frac{\pi }{2}-\frac{\theta_{i}}{2}-\theta_{e}\right)}$$

#### 4.2.2 Coordinate conversion of the auxiliary navigation information

Through the joint calibration of the camera and LiDAR in the above section, the camera external parameter matrix (*R, T*), the camera internal parameter, and the rigid conversion matrix (*R_lidar_*, *T_lidar_*), of the camera and LiDAR sensor information were obtained.

In order to supplement the main navigation information with the auxiliary navigation information, it is essential to establish a conversion model between sensors. Through the established transformation model, the points in the world coordinate system scanned by LiDAR were projected into the pixel coordinate system of the camera to realize the supplementation of the point cloud data to the visual information according to the pinhole camera model, as shown in **Figure 7B**. Note that, in **Figure 7B**, *P* is the point on the real object, *p* is the imaging point of *P* in the image, (*x, y*) are the coordinates of *p* in the image coordinate system, (*u, v*) are the coordinates of *p* in the pixel coordinate system, and *f* is the focal length of the camera, where *f* = || *o* – *0*_c_|| (in millimeters). The corresponding relationship between a point *P*(*X_w_, Y_w_, Z_w_*) in the real world obtained by LiDAR and the corresponding point *p*(*u, v*) in the camera pixel coordinate system can be expressed as:


(11) $$\left[\begin{array}{c} u\\ v\\ 1 \end{array}\right]=\left[\begin{array}{ccc} \begin{array}{c} f_{x}\\ 0\\ 0 \end{array} & \begin{array}{c} 0\\ f_{y}\\ 0 \end{array} & \begin{array}{cc} \begin{array}{c} u_{0}\\ v_{0}\\ 1 \end{array} & \begin{array}{c} 0\\ 0\\ 0 \end{array} \end{array} \end{array}\right]\left[\begin{array}{cc} \begin{array}{c} R_{lidar}\\ 0^{T} \end{array} & \begin{array}{c} T_{lidar}\\ 1 \end{array} \end{array}\right]\left[\begin{array}{c} X_{w}\\ Y_{w}\\ \begin{array}{c} Z_{w}\\ 1 \end{array} \end{array}\right]$$

According to the principle of LiDAR scanning, the point cloud data obtained by LiDAR are in the form of polar coordinates. Therefore, the distance and angle information of the point cloud data under polar coordinates were converted into the three-dimensional coordinate point information under the LiDAR ontology coordinate system. The conversion formula was as follows:


(12) $$\{\begin{array}{c} X_{w}=\rho \cdot cos\alpha \cdot cos\theta \\ Z_{w}=\rho \cdot cos\alpha \cdot sin\theta \\ Y_{w}=\rho \cdot sin\alpha \end{array}$$

where *ρ* is the distance between the scanning point and the LiDAR;*α* is the elevation angle of the scanning line at the scanning point, namely, the angle in the vertical direction; and θ is the heading angle in the horizontal direction.

In order to eliminate the camera imaging distortion error caused by the larger deflection of light away from the lens center and the lens not being completely parallel to the image plane, as shown in **Figure 7C**, we corrected the distortion of Equation (11)with the correction formula, given as follows (Chen et al., 2021b):

Radial distortion correction:


(13) $$\{\begin{array}{c} u'=u(1+k_{1}r^{2}+k_{2}r^{4})\\ v'=v(1+k_{1}r^{2}+k_{2}r^{4}) \end{array}$$

Tangential distortion correction:


(14) $$\{\begin{array}{c} u''=u'+(2p_{1}v'+p_{2}(r^{2}+2u{'}^{2})\\ v''=v'+(2p_{2}u'+p_{1}(r^{2}+2v{'}^{2}) \end{array}$$

Where *k*_1_ and *k*_2_ are the radial correction parameters; *p*_1_ and *p*_2_ are the tangential correction parameters; *u*′′and *v*′ re the radially corrected pixel coordinates; and *u*′′ and *v′′* are the tangentially corrected pixel coordinates.

The corresponding relationship between the point in the world coordinate system obtained by LiDAR and the camera pixel coordinate system is established through Equations (10)–(14). According to the established coordinate transformation model, the LiDAR point cloud data can be converted to the image space for the purpose of supplementation between machine vision and LiDAR.

#### 4.2.3 Feature recognition of point cloud based on PointNet

**Figure 8:**
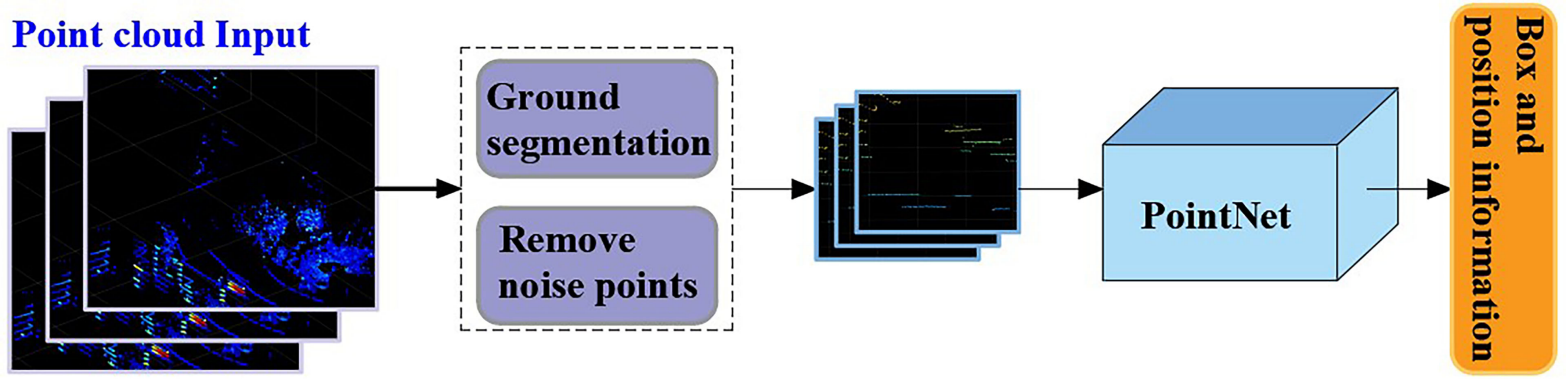
The principle of auxiliary navigation.

Because of the irregular format of the point cloud, it is difficult to extract its feature, but with the proposal of the PointNet model (Jing et al., 2021), this problem was solved. In this paper, the features of the non-maize obstacles in the middle and late stages of maize were extracted through PointNet, and their location information taken as the output. Note that we also performed the following work before using the PointNet model for training. The principle is shown in **Figure 8**.

##### 4.2.3.1 Ground segmentation

In order to obtain auxiliary navigation information from the LiDAR point cloud data, the ground point cloud must be segmented first. In this work, the RANSAC (random sample consensus) algorithm was adopted to segment the collected point cloud data.

The unique plane can be determined by randomly selecting three non-collinear sample points (*x_a_, x_b_, x_c_*) in the point cloud.


(15) $$n_{i}\cdot x+d_{i}=0$$

(16) $$n_{i}=(x_{b}-x_{a})(x_{c}-x_{a})$$

(17) $$d_{i}=-n\cdot x_{a}$$

Where *n_i_* is the normal vector of the plane model and *d_i_* is the pitch of the plane model. Then, the distance from any sample point *x_i_* in the point cloud to the plane model is given by


(18) $$r_{i}={\left(n_{i}\cdot x_{i}+d_{i}\right)}^{2}$$

Let the distance threshold be *T*, when *r_i_<T*. The sample point *x_i_* is the internal point; otherwise, it is the external point. Let *N* be the number of internal points with


(19) $$N=NUM(x_{i}),r_{i}<T$$

sNote that Equations (15)–(19) show a calculation process, but *N* is not necessarily the maximum value at this time; hence, an iterative calculation is needed. Let the number of iterations be *k_c_*. When *N* takes the maximum value, *N*_max_, in the iterative process, the plane model corresponding to *n*_best_ and *d*_best_ is the best-fitting ground.

##### 4.2.3.2 Removing noise points caused by maize leaves

LiDAR was mainly used to identify obstacles other than maize leaves. In order to reduce the difficulty of model training, the point cloud data of maize leaves were deleted. This technology depends on the analysis of the *z*-coordinate distribution of each point cloud. In general, the height of obstacles such as soil blocks and stones is less than 10 cm. Therefore, when we trained the model sexually, we deleted the point cloud with a *z*-coordinate greater than 10 cm in the *θ_e_* range.

## 5 Experiments and discussions

The focus of this paper was navigation information acquisition. Navigation information can be used for path planning to guide the robot to drive autonomously and can also be used as the basis for the adjustment of the driving state of the robot, such as reducing the driving speed when detecting rocks or large clods. We provided the results of the information acquisition experiment.

### 5.1 Main navigation information acquisition experiment

We verified the recognition performance of the Im-YOLOv5 for the main navigation information from two aspects: model training and detection results. In order to facilitate comparisons, we also provided the test results of YOLOv5 and Faster-RCNN (faster region-based convolutional network). The datasets used in the experiment were collected by the Anhui Intelligent Agricultural Machinery Equipment Engineering Laboratory. It should be noted that, in order for each model to perform best on the datasets, we adjusted the parameters of each model separately to select the appropriate hyperparameters. The initial hyperparameter settings of each algorithm are shown in **Table 2**. We divided the train set, test set, and verification set according to an 8:1:1 ratio, and the dataset contained 3,000 images.

**Table 2:** Target detection hyperparameter setting.

Parameter	Im-YOLOv5	YOLOv5	Faster-RCNN
Backbone network	MobileNetv2	Backbone	Resnet50
Training size	640 × 640	640 × 640	640 × 640
Batch size	16	16	8
No. of categories	5	5	5
Initial learning rate	1e−2	1e−2	1e−4
No. of iterations	100	100	100

Im-YOLOv5, improved You Only Look Once, version 5; Faster-RCNN, faster region-based convolutional network.

**Figure 9:**
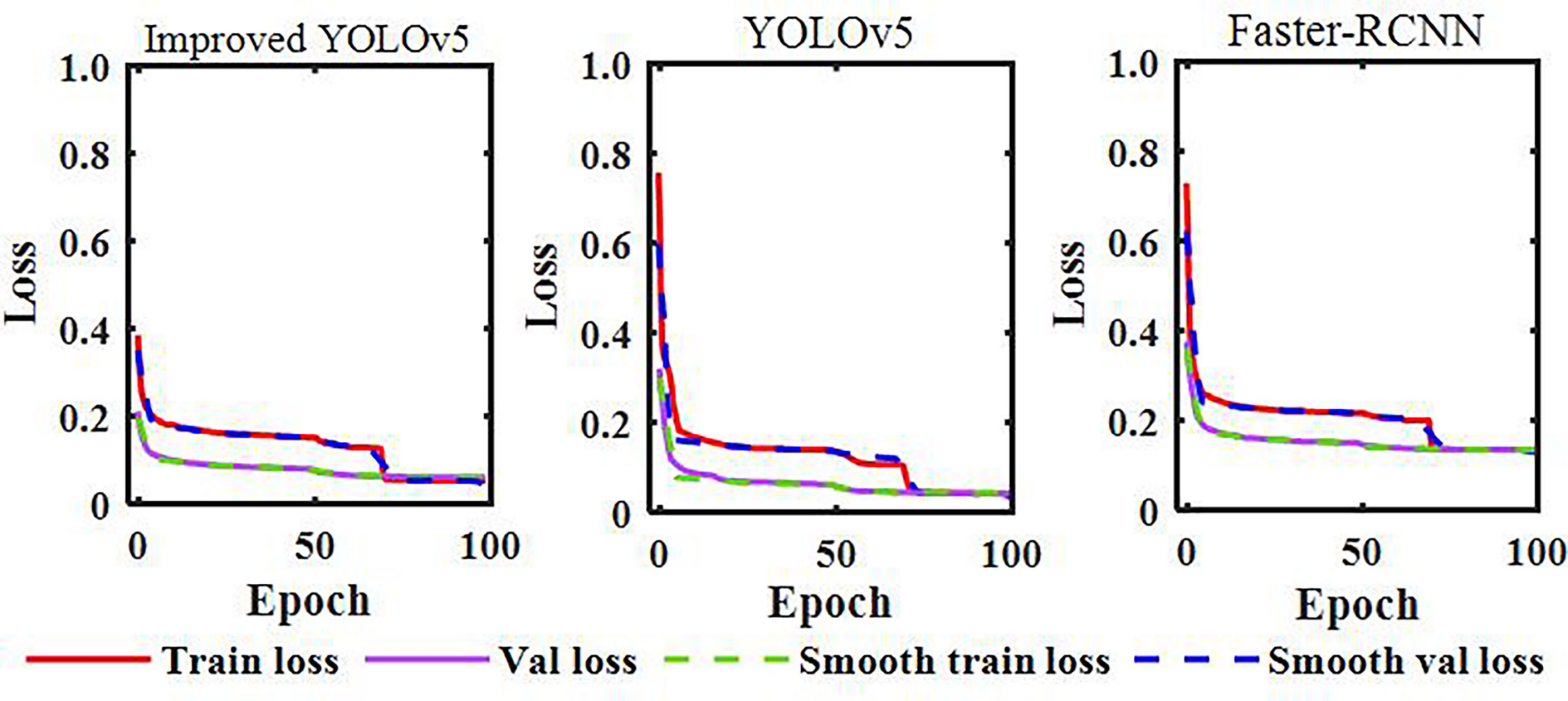
Model training and validation loss rate curves.

The model training and validation loss rate curves are shown in **Figure 9**. From the figure, it can be seen that the loss rate tends to stabilize with the increase of iterations, finally converging to the fixed value; this indicates that the model has reached the optimal effect. The debugged model showed good fitting and generalization ability for the maize stem datasets. Note that, due to the Im-YOLOv5 having an improved loss function, the initial loss value of the model was about 0.38, which was the lowest among the three models, and the convergence speed was accelerated.

**Figure 10:**
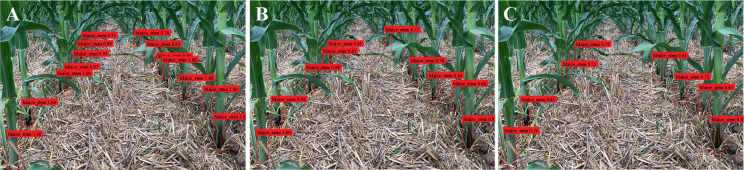
Results of stem detection. **(A)** Improved You Only Look Once, version 5 algorithm (Im-YOLOv5). **(B)** YOLOv5. **(C)** Faster region-based convolutional network (Faster-RCNN).

The *P* (comparison of accuracy), *R* (recall), *F*_1_ (harmonic average), FPS (frame rate), and mAP (mean average precision) values for Im-YOLOv5, YOLOv5, and Faster-RCNN are shown in **Table 3**. From the table, it can be seen that Im-YOLOv5 had the highest accuracy rate, followed by YOLOv5; the accuracy rate of Faster-RCNN was low. With the lightweight backbone network, the FPS of Im-YOLOv5 was the highest, and the weight was greatly reduced. While meeting the real-time requirements, the detection speed of a single image was also the fastest and the detection performance was the best. Compared with that of YOLOv5, the FPS of Im-YOLOv5 was increased by 17.91% and the model size reduced by 55.56% when the mAP was reduced by only 0.35%, which improved the detection performance and shortened the model reasoning time. From the datasets, we selected a number of inter-row images of maize in the middle and late stages for testing, as shown in **Figure 10**. For the same image, Im-YOLOv5 was able to identify most maize stems, even those that were partially covered. At the same time, the detection confidence of Im-YOLOv5 and YOLOv5 was high, but that of Faster-RCNN was relatively low.

**Table 3:** Model evaluation.

Model	*P* (%)	*R* (%)	*F*_1_ (%)	FPS	Model size (M)	mAP (%)
Im-YOLOv5	97	81	88	78	12	96.12
YOLOv5	93	90	93	66	27	96.48
Faster-RCNN	76	92	82	20	108	90.52

P, comparison of accuracy; R, recall; F_1_, harmonic average; FPS, frame rate; mAP, mean average precision.

### 5.2 Auxiliary navigation information supplements the main navigation information experiment

**Figure 11:**
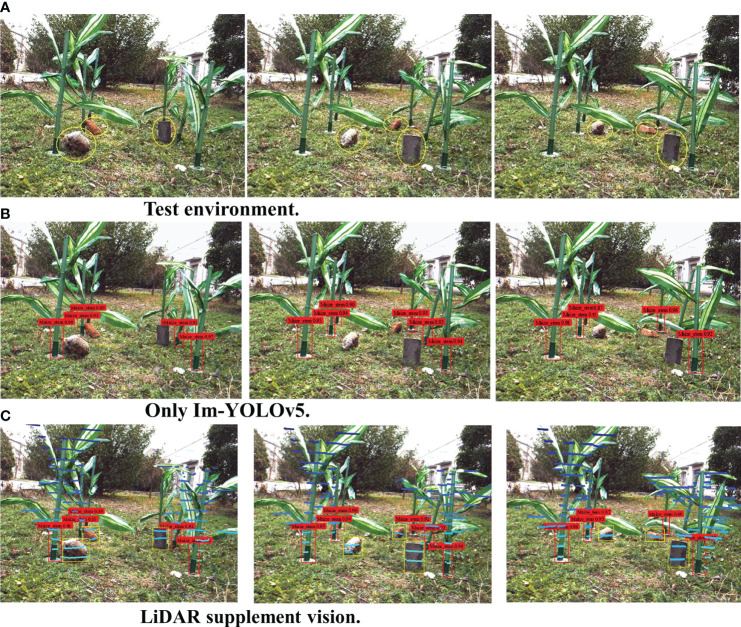
**(A)** Test environment. **(B)** Only the improved You Only Look Once, version 5 algorithm (Im-YOLOv5). **(C)** Laser imaging, detection, and ranging (LiDAR) supplement vision.

In the experiments, the practical feasibility of the proposed inter-row navigation information acquisition method was verified based on LiDAR point cloud data-supplemented machine vision in the middle and late stages of maize. Considering the current coronavirus outbreak, conducting large-scale field experiments had been difficult. Therefore, an artificial maize plant model was used to set up the simulation test environment for verifying the feasibility of the designed method. **Figure 11A** shows the test environment using the maize plant model. Investigation of maize planting in Anhui Province revealed that the row spacing for maize plants is about 50–80 cm and that plant spacing is about 20–40 cm. Therefore, the row spacing in the maize plant model was set to 65 cm and the plant spacing to 25 cm. At the same time, a number of non-maize obstacles were also set in the experiments. For the purpose of data acquisition in this work, the data acquisition robot was developed by Anhui Intelligent Agricultural Machinery and Equipment Engineering Laboratory at Anhui Agricultural University.

During the experiments, the required main navigation information was the position information of maize plants, while the required auxiliary navigation information was the position information of the non-maize obstacles. We set up six maize plant models and three non-maize obstacles and randomly set the locations of the obstacles. Subsequently, we conducted 10 information acquisition experiments at distances of 1,000, 2,000, and 3,000 mm from the data acquisition robot to the front row of the maize plant model. The test results are shown in **Figures 11B, C**.

### 5.3 Discussions

Generally, visual navigation between rows in the middle and late stages of maize extracts the maize characteristics and then fits the navigation path. If the camera was only used to obtain information based on the maize characteristics in the recognition stage, information on the non-maize obstacles between rows in the middle and late stages of maize is missed, as shown in **Figures 11B, C**. With the introduction of the Im-YOLOv5 stem recognition algorithm, sufficient training for maize stem recognition has become exceptionally accurate; however, the non-maize obstacle recognition rate was almost zero only for Im-YOLOv5, which is extremely fatal for the actual operation safety of plant protection robots in the middle and late stages of maize.

When using LiDAR to obtain auxiliary navigation information in order to supplement the main navigation information obtained by machine vision, the issue of missing information can be properly solved, with the safety of the planned navigation path under this condition being greatly improved. However, due to the recognition accuracy of the 16-line LiDAR and the error of the camera–LiDAR joint calibration, the information recognition effect was not very satisfactory when the obstacle is far away and is too small. With increasing distance between the data acquisition robot and the maize plant, the number of maize plant models can be stably maintained, which means that the identification of the main navigation information is also stable. However, recognition of the number of non-maize obstacles showed a downward trend, indicating that the recognition accuracy using the auxiliary navigation information was reduced. In view of these issues, we will be using the 32-line or the 64-line LiDAR, both with higher accuracy, in future experiments.

## 6 Conclusion

In order to solve the problem of missing information when using machine vision for inter-row navigation in the middle and late stages of maize, this paper has proposed a method using LiDAR point cloud data to supplement machine vision in order to obtain more accurate inter-row information in the middle and late stages of maize. Through training of the machine vision datasets with the Im-YOLOv5 model, the main navigation information was obtained by identifying maize plants between the rows of maize in the middle and late stages. As a supplement to the main navigation information acquired by machine vision, LiDAR has been used to provide additional information to identify information on other non-crop obstacles as auxiliary navigation information. Not only was the accuracy of information recognition improved, but technical support for planning a safe navigation path can also be provided. Experimental results from the data acquisition robot equipped with a camera and a LiDAR sensor have demonstrated the validity and the good inter-row navigation recognition performance of the proposed method for the middle and late stages of maize. However, with the improvement in the accuracy of LiDAR, cost is the key problem restricting the commercialization of this recognition system. Therefore, we hope that our recognition system can be applied in small autonomous navigation plant protection robots, as the relatively low cost of small plant protection robots, even with the application of this relatively high-precision recognition system, has a price advantage over UAVs. The navigation information can be used for path planning to guide robots to drive autonomously and can also be used as the basis for the adjustment of the driving state of robots, such as in reducing the driving speed when detecting rocks or large clods. Therefore, in subsequent research, we will focus on path planning between maize rows and the control of the driving state of robots.

## Data availability statement

The raw data supporting the conclusions of this article will be made available by the authors, without undue reservation.

## Author contributions

ZL: Software, visualization, investigation, and writing—original draft. DX and LL: Investigation. HW: Writing—review and editing. LC: Conceptualization, methodology, writing—review and editing. All authors contributed to the article and approved the submitted version.

## Funding

This work was supported in part by the National Natural Science Foundation of China under grant no. 52175212.

## Conflict of interest

The authors declare that the research was conducted in the absence of any commercial or financial relationships that could be construed as a potential conflict of interest.

## Publisher’s note

All claims expressed in this article are solely those of the authors and do not necessarily represent those of their affiliated organizations, or those of the publisher, the editors and the reviewers. Any product that may be evaluated in this article, or claim that may be made by its manufacturer, is not guaranteed or endorsed by the publisher.

## References

[B1] Aguiar A. Oliveira M. Pedrosa E. F. Santos F. (2021). A camera to LiDAR calibration approach through the optimization of atomic transformations. Expert Syst. Appl. 176, 114894. doi: 10.1016/j.eswa.2021.114894

[B2] Bae H. Lee G. Yang J. Shin G. Lim Y. Choi G. (2021). Estimation of closest in-path vehicle (CIPV) by low-channel LiDAR and camera sensor fusion for autonomous vehicle. Sensors. 21, 3124. doi: 10.3390/s21093124 33946282 PMC8125378

[B3] Chen X. Chen Y. Song X. Liang W. Wang Y. (2021a). Calibration of stereo cameras with a marked-crossed fringe pattern. Opt. Lasers Eng. 147, 106733. doi: 10.1016/j.optlaseng.2021.106733

[B4] Chen K.-W. Lai C.-C. Lee P.-J. Chen C.-S. Hung Y.-P. (2011). Adaptive learning for target tracking and true linking discovering across multiple non-overlapping cameras. IEEE Trans. Multimedia 13, 625–638. doi: 10.1109/TMM.2011.2131639

[B5] Chen L. Li Z. Yang J. Song Y. (2021). Lateral stability control of four-Wheel-Drive electric vehicle based on coordinated control of torque distribution and ESP differential braking. Actuators 10, 135. doi: 10.3390/act10060135

[B6] Chen X. Song X. Cheng L. Ran Z. Wang Y. Liang W.. (2021b). Flexible calibration method of electronically focus-tunable lenses. IEEE Trans. Instrum. Meas 70, 5013210. doi: 10.1109/TIM.2021.3097412

[B7] Chen L. Wang P. Zhang P. Zheng Q. He J. Wang Q. (2018). Performance analysis and test of a maize inter-row self-propelled thermal fogger chassis. Int. J. Agric. Biol. Eng. 11, 100–107. doi: 10.25165/j.ijabe.20181105.3607

[B8] Gai J. Xiang L. Tang L. (2021). Using a depth camera for crop row detection and mapping for under-canopy navigation of agricultural robotic vehicle. Comput. Electron. Agric. 188, 106301. doi: 10.1016/j.compag.2021.106301

[B9] Gu Y. Li Z. Zhang Z. Li J. Chen L. (2020). Path tracking control of field information-collecting robot based on improved convolutional neural network algorithm. Sensors 20, 797. doi: 10.3390/s20030797 32024030 PMC7038679

[B10] Hassanin M. Anwar S. Radwan I. Khan F. S. Mian A. (2022). Visual attention methods in deep learning: An in-depth survey. arXiv. doi: 10.48550/arXiv.2204.07756

[B11] Hiremath S. A. van der Heijden G. W. A. M. van Evert F. K. Stein A. ter Braak C. J. F. (2014). Laser range finder model for autonomous navigation of a robot in a maize field using a particle filter. Comput. Electron. Agric. 100, 41–50. doi: 10.1016/j.compag.2013.10.005

[B12] Jafari Malekabadi A. Khojastehpour M. Emadi B. (2019). Disparity map computation of tree using stereo vision system and effects of canopy shapes and foliage density. Comput. Electron. Agric. 156, 627–644. doi: 10.1016/j.compag.2018.12.022

[B13] Jeong J. Yoon T. S. Park J. B. (2018). Multimodal sensor-based semantic 3D mapping for a Large-scale environment. Expert Syst. Appl. 105, 1–10. doi: 10.1016/j.eswa.2018.03.051

[B14] Jing Z. Guan H. Zhao P. Li D. Yu Y. Zang Y.. (2021). Multispectral LiDAR point cloud classification using SE-PointNet plus. Remote Sens. 13, 2516. doi: 10.3390/rs13132516

[B15] Jubayer F. Soeb J. A. Mojumder A. N. Paul M. K. Barua P. Kayshar S.. (2021). Detection of mold on the food surface using YOLOv5. Curr. Res. Food Sci. 4, 724–728. doi: 10.1016/j.crfs.2021.10.003 34712960 PMC8529025

[B16] Li Z. Chen L. Zheng Q. Dou X. Yang L. (2019). Control of a path following caterpillar robot based on a sliding mode variable structure algorithm. Biosyst. Eng. 186, 293–306. doi: 10.1016/j.biosystemseng.2019.07.004

[B17] Liu L. Mei T. Niu R. Wang J. Liu Y. Chu S. (2016). RBF-based monocular vision navigation for small vehicles in narrow space below maize canopy. Appl. Sci.-Basel 6, 182. doi: 10.3390/app6060182

[B18] Liu H. Yao Y. Sun Z. Li X. Jia K. Tang Z. (2020). Road segmentation with image-LiDAR data fusion in deep neural network. Multimed. Tools Appl. 79, 35503–35518. doi: 10.1007/s11042-019-07870-0

[B19] Morales J. Vázquez-Martín R. Mandow A. Morilla-Cabello D. García-Cerezo A. (2021). The UMA-SAR dataset: Multimodal data collection from a ground vehicle during outdoor disaster response training exercises. Int. J. Robotics Res. 40, 27836492110049. doi: 10.1177/02783649211004959

[B20] Mutz F. W. Oliveira-Santos T. Forechi A. Komati K. S. Badue C. Frana F.. (2021). What is the best grid-map for self-driving cars localization? an evaluation under diverse types of illumination, traffic, and environment. Expert Syst. Appl. 179, 115077. doi: 10.1016/J.ESWA.2021.115077

[B21] Patricio D. I. Rieder R. (2018). Computer vision and artificial intelligence in precision agriculture for grain crops: A systematic review. Comput. Electron. Agric. 153, 69–81. doi: 10.1016/j.compag.2018.08.001

[B22] Radcliffe J. Cox J. Bulanon D. M. (2018). Machine vision for orchard navigation. Comput. Ind. 98, 165–171. doi: 10.1016/j.compind.2018.03.008

[B23] Reiser D. Vázquez-Arellano M. Paraforos D. S. Garrido-Izard M. Griepentrog H. W. (2018). Iterative individual plant clustering in maize with assembled 2D LiDAR data. Comput. Industry 99, 42–52. doi: 10.1016/j.compind.2018.03.023

[B24] Tang Y. Chen M. Wang C. Luo L. Li J. Lian G.. (2020). Recognition and localization methods for vision-based fruit picking robots: A review. Front. Plant Sci. 11. doi: 10.3389/fpls.2020.00510 PMC7250149 32508853

[B25] Tang D. Feng Y. Gong D. Hao W. Cui N. (2018). Evaluation of artificial intelligence models for actual crop evapotranspiration modeling in mulched and non-mulched maize croplands. Comput. Electron. Agric. 152, 375–384. doi: 10.1016/j.compag.2018.07.029

[B26] Tang Y. Zhou H. Wang H. Zhang Y. (2023). Fruit detection and positioning technology for a camellia oleifera c. Abel orchard based on improved YOLOv4-tiny model and binocular stereo vision. Expert Syst. Appl. 211, 118573. doi: 10.1016/j.eswa.2022.118573

[B27] Wang Y. Cai J. Liu Y. Chen X. Wang Y. (2022a). Motion-induced error reduction for phase-shifting profilometry with phase probability equalization. Opt. Lasers Eng. 156, 107088. doi: 10.1016/j.optlaseng.2022.107088

[B28] Wang Y. Cai J. Zhang D. Chen X. Wang Y. (2022b). Nonlinear correction for fringe projection profilometry with shifted-phase histogram equalization. IEEE Trans. Instrum. Meas 71, 5005509. doi: 10.1109/TIM.2022.3145361

[B29] Wang H. Lin Y. Xu X. Chen Z. Wu Z. Tang Y. (2022). A study on long-close distance coordination control strategy for litchi picking. Agronomy-Basel 12, 1520. doi: 10.3390/agronomy12071520

[B30] Wang Y. Wen W. Wu S. Wang C. Yu Z. Guo X.. (2018). Maize plant phenotyping: Comparing 3D laser scanning, multi-view stereo reconstruction, and 3D digitizing estimates. Remote Sens. 11, 63. doi: 10.3390/rs11010063

[B31] Wu F. Duan J. Chen S. Ye Y. Ai P. Yang Z. (2021). Multi-target recognition of bananas and automatic positioning for the inflorescence axis cutting point. Front. Plant Sci. 12. doi: 10.3389/fpls.2021.705021 PMC8592935 34795680

[B32] Xie L. Xu Y. Zhang X. Bao W. Tong C. Shi B. (2019). A self-calibrated photo-geometric depth camera. Visual Comput. 35, 99–108. doi: 10.1007/s00371-018-1507-9

[B33] Xue H. Sun M. Liang Y. (2022). ECANet: Explicit cyclic attention-based network for video saliency prediction. Neurocomputing 468, 233–244. doi: 10.1016/j.neucom.2021.10.024

[B34] Xu X. Zhang L. Yang J. Liu C. Xiong Y. Luo M.. (2021). LiDAR-camera calibration method based on ranging statistical characteristics and improved RANSAC algorithm. Robot. Auton. Syst. 141, 103776. doi: 10.1016/j.robot.2021.103776

[B35] Xu X. Zhuge S. Guan B. Lin B. Gan S. Yang X.. (2022). On-orbit calibration for spaceborne line array camera and LiDAR. Remote Sens. 14, 2949. doi: 10.3390/rs14122949

[B36] Yang T. Bai Z. Li Z. Feng N. Chen L. (2021). Intelligent vehicle lateral control method based on feedforward. Actuators 10, 228. doi: 10.3390/act10090228

[B37] Yang Y. Li Y. Wen X. Zhang G. Ma Q. Cheng S.. (2022a). An optimal goal point determination algorithm for automatic navigation of agricultural machinery: Improving the tracking accuracy of the pure pursuit algorithm. Comput. Electron. Agric. 194, 106760. doi: 10.1016/j.compag.2022.106760

[B38] Yang Z. Ouyang L. Zhang Z. Duan J. Yu J. Wang H. (2022b). Visual navigation path extraction of orchard hard pavement based on scanning method and neural network. Comput. Electron. Agric. 197, 106964. doi: 10.1016/j.compag.2022.106964

[B39] Yu J. Ma L. Tian M. Lu X. (2021). Registration and fusion of UAV LiDAR system sequence images and laser point clouds. J. Imaging Sci. Technol. 65, 10501. doi: 10.2352/J.ImagingSci.Technol.2021.65.1.010501

[B40] Zhang Z. Jia X. Yang T. Gu Y. Wang W. Chen L. (2021). Multi-objective optimization of lubricant volume in an ELSD considering thermal effects. Int. J. Therm. Sci. 164, 106884. doi: 10.1016/j.ijthermalsci.2021.106884

[B41] Zhang Z. Li P. Zhao S. Lv Z. An Y. (2020). An adaptive vision navigation algorithm in agricultural IoT system for smart agricultural robots. Computers Mater. Continua 66, 1043–1056. doi: 10.32604/cmc.2020.012517

[B42] Zhou Z. Song Z. Fu L. Gao F. Li R. Cui Y. (2020). Real-time kiwifruit detection in orchard using deep learning on android (TM) smartphones for yield estimation. Comput. Electron. Agric. 179, 105856. doi: 10.1016/j.compag.2020.105856

